# Nucleosomes at the Dawn of Eukaryotes

**DOI:** 10.1093/gbe/evae029

**Published:** 2024-02-14

**Authors:** Antoine Hocher, Tobias Warnecke

**Affiliations:** Medical Research Council Laboratory of Medical Sciences, London, UK; Institute of Clinical Sciences, Faculty of Medicine, Imperial College London, London, UK; Medical Research Council Laboratory of Medical Sciences, London, UK; Institute of Clinical Sciences, Faculty of Medicine, Imperial College London, London, UK; Trinity College, University of Oxford, Oxford, UK

**Keywords:** chromatin, histones, nucleosome, evolution, archaea, Asgardarchaea

## Abstract

Genome regulation in eukaryotes revolves around the nucleosome, the fundamental building block of eukaryotic chromatin. Its constituent parts, the four core histones (H3, H4, H2A, H2B), are universal to eukaryotes. Yet despite its exceptional conservation and central role in orchestrating transcription, repair, and other DNA-templated processes, the origins and early evolution of the nucleosome remain opaque. Histone-fold proteins are also found in archaea, but the nucleosome we know—a hetero-octameric complex composed of histones with long, disordered tails—is a hallmark of eukaryotes. What were the properties of the earliest nucleosomes? Did ancestral histones inevitably assemble into nucleosomes? When and why did the four core histones evolve? This review will look at the evolution of the eukaryotic nucleosome from the vantage point of archaea, focusing on the key evolutionary transitions required to build a modern nucleosome. We will highlight recent work on the closest archaeal relatives of eukaryotes, the Asgardarchaea, and discuss what their histones can and cannot tell us about the early evolution of eukaryotic chromatin. We will also discuss how viruses have become an unexpected source of information about the evolutionary path toward the nucleosome. Finally, we highlight the properties of early nucleosomes as an area where new tools and data promise tangible progress in the not-too-distant future.

**Significance**

The nucleosome lies at the heart of eukaryotic genome regulation. Its individual components, the four core histones, are universal to (and nucleosome architecture is invariant across) eukaryotes. In this review, we summarize our current knowledge of how this critical histone–DNA complex emerged during early eukaryotic evolution, focusing on recent data from the closest archaeal relatives of eukaryotes, the Asgardarchaea. We discuss critical evolutionary transitions required to evolve modern nucleosomes and highlight to what extent precursors for key features of the nucleosome—conserved paralogs, obligate heterodimerization, and lysine-rich tails—can be found in the archaeal ancestors of eukaryotes.

## Main

The genomes of all organisms are associated with proteins that package and protect them (Luijsterburg et al. 2008). These proteins also regulate, to varying degrees, access to the underlying genetic information.

In eukaryotes, most nuclear DNA is wrapped around a conserved octameric complex composed of four different histone proteins (H3, H4, H2A, and H2B). This protein–DNA complex, the nucleosome, is the fabric of eukaryotic chromatin and lies at the very heart of eukaryotic genome regulation. Like many nucleoid-associated proteins (NAPs) in prokaryotes, histones constrain negative supercoils and contribute to genome compaction. But, in the context of the nucleosome, histones do a great deal more. Notably, nucleosomes generate a restrictive transcriptional ground state, where chromatin remodeling is required for gene activation (Struhl 1999; Madhani 2013). In addition, they orchestrate dynamic access to the genome, through a bewildering array of posttranslational modifications (PTMs), which are deposited, recognized, and removed by numerous dedicated enzymes. Together, these PTMs can reinforce restrictive or generate permissive chromatin states that allow DNA-templated processes like transcription, replication, and DNA repair to proceed (Khorasanizadeh 2004). While the nucleosome may not *define* eukaryotic genome regulation, the latter is hard to imagine without the former.

How did the nucleosome come to occupy this critical position in eukaryotic genome regulation? What are the origins of the complex, highly modified and indispensable nucleosomes we know today? What did the nucleosome of the last eukaryotic common ancestor (LECA) look like? What properties did it possess? Did it restrict access to DNA in the same manner as present-day nucleosomes? Was its primordial function in gene regulation or elsewhere? Did ancestral histones carry tails? If so, what functions did those tails serve? Were early eukaryotic histones already the subject of PTM? And did they, as previously suggested (Brunk and Martin 2019), play a critical role in eukaryogenesis, facilitating both genome size expansion and regulatory diversification?

This review will highlight recent progress in addressing some of the questions above and point out persistent gaps in our knowledge. We will focus specifically on features of the nucleosome that set it apart from archaeal histone–DNA complexes and the key transitions required to evolve the nucleosome as we know it.

### Deep Conservation of the Eukaryotic Nucleosome

**Fig. 1.**
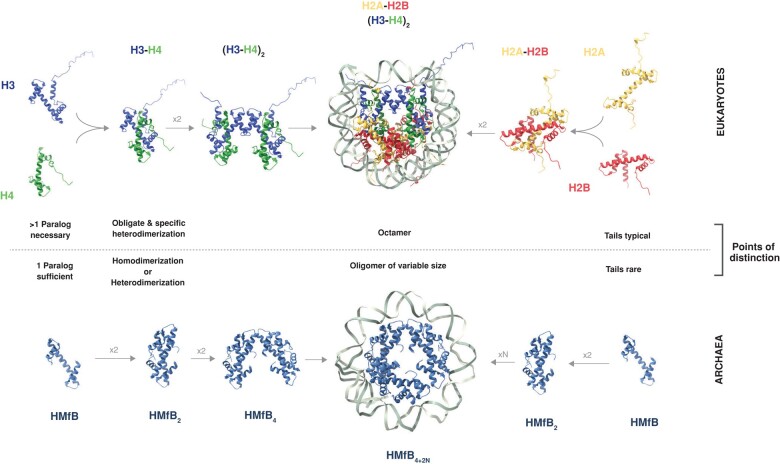
Histone–DNA complexes in eukaryotes and archaea. Schematic of the assembly of eukaryotic nucleosomes (top, illustrated using PDB 1AOI from *Xenopus laevis*) and model archaeal hypernucleosomes (bottom, illustrated using PDB 5T5K from *Methanothermus fervidus*) from their constituent histone monomers, highlighting salient differences between the two.

The nucleosome contains an octameric protein complex, assembled from two copies each of four ancient histone paralogs: H3, H4, H2A, and H2B (Fig. 1). These four core histones share a central histone-fold domain but can be distinguished based on characteristic substitutions and structural features, including N- and C-terminal extensions, which are often referred to as tails (Luger et al. 1997; Luger and Richmond 1998a, 1998b). H3 and H4 form an obligate heterodimer, as do H2A and H2B. Two H3–H4 dimers form a tetramer on DNA, and two H2A–H2B dimers assemble onto this scaffold, enabling further wrapping of DNA to yield the final complex, which contacts ∼150 bp of DNA (Luger et al. 1997). Consecutive nucleosomes along the genome are connected by stretches of linker DNA of variable length, which do not contact the core histones but can be bound by the “linker histone”, H1, which does not contain a histone-fold domain and has a distinct evolutionary history (Kasinsky et al. 2001).

Assembly and disassembly of nucleosomes is dynamic, and can involve the exchange of core replicative histones for specific variants (e.g. H3.3, H2A.Z) that endow the nucleosome with different biophysical properties or affect which proteins will be recruited as interactors (Henikoff and Smith 2015). The overall assembly plan, however, is invariant across eukaryotes. The position of individual histones within the complex is fixed, as is its overall size. (There are, of course, exceptions: assembly intermediates naturally contain fewer than eight histones and there are variant complexes such as hemisomes, which assemble on centromeric DNA (Dalal et al. 2007), hexasomes (Baer and Rhodes 1983), and telomeric nucleosomes, where octamers are present but stacked in direct contact with each other to form larger contiguous histone–DNA complexes (Soman et al. 2022). These exceptions, however, are typically rare in number and exist alongside the classic, hetero-octameric nucleosomes, which make up the bulk of eukaryotic chromatin.)

Ordinarily, to understand the evolution of a protein complex and how its structure and function have changed over time, one might survey extant genomes for members of that complex and then ask which subunits of the complex have been added or lost along different lineages, hoping to find a stepwise path to complexity, reconstruct informative evolutionary intermediates, and discover variation that illustrates the evolutionary malleability of the complex. In addition, reconstructing changes that occurred in individual members of the complex can reveal its ancestral capacities and functional evolution (Finnigan et al. 2012; Perica et al. 2014; Reisinger et al. 2014). At the extreme, the entire complex might be absent in some organisms, and this might point to the conditions under which the complex becomes dispensable.

The nucleosome is striking in its lack of cross-species variation. Yes, core histones have duplicated and diverged along different eukaryotic lineages into a plethora of variants that can be swapped into and diversify the properties of the nucleosome (see Henikoff and Smith (2015) for a comprehensive review). But nucleosome *architecture* appears invariant across eukaryotes. Support for this comes not only from genome inventories, which consistently recover all four core histones, but also from crystal structures of nucleosomes from divergent eukaryotes, including the kinetoplastid *Trypanosoma brucei* (PDB ID: 8COM) and the metamonad *Giardia intestinalis* (PDB ID: 7D69), along with structures from humans (e.g. PDB ID: 6R93), flies (e.g. PDB ID: 2PYO), and fungi (e.g. PDB ID: 1ID3, 7WLR). Granted, these structures show some differences in complex stability and how tightly DNA is wrapped (Sato et al. 2021; Deák et al. 2023), but overall architecture is highly conserved—a case of animated stasis rather than evidence for major evolutionary transitions. Some histones, notably from dinoflagellates (Marinov and Lynch 2015) and nucleomorph genomes (Marinov and Lynch 2016), are unusually divergent and might give rise to nucleosomes with more radically different properties, but even they come in the conserved set of four core histones and therefore likely assemble complexes with the same basic architecture. In fact, the four core histones are universal to eukaryotes; there are no verified cases where even one of the four has been lost in any eukaryotic genome examined to date (Soo and Warnecke 2021). By implication, the nucleosome as a hetero-octameric complex already existed at the time of LECA.

This lack of variation amongst eukaryotes forces one to peer deeper into the past to understand the origins of the nucleosome.

### Archaeal Histones—So Near and Yet So Far

Eukaryotes are the product of a merger between archaea and bacteria, with the latter evolving into mitochondria and plastids (López-García and Moreira 2023). Although histone-fold proteins can be found in several bacteria (Hocher et al. 2023), eukaryotic histones most probably have archaeal ancestry, like many other cellular components involved in information processing (Malik and Henikoff 2003; Koonin 2015). Structural analysis of histones from model archaea has revealed remarkable conservation at different levels of organization, from the core histone fold and antiparallel dimerization (Decanniere et al. 2000) to residue-level contacts between histones and DNA and the formation of tetramers that bind and bend a ∼60 bp piece of DNA (Mattiroli et al. 2017).

Unlike their distant eukaryotic cousins, however, histones from model archaea like *Thermococcus kodakarensis* are not limited to specific heterodimerization partners; they readily homo- as well as heterodimerize (reviewed in Stevens and Warnecke (2022)), a feature that might be common beyond model species (Stevens et al. 2020). Archaeal dimers also go on to form higher order complexes; but whereas archaeal tetramers exhibit a very similar architecture to (H3–H4)_2_ tetramers found in eukaryotes, they do not nucleate nucleosomes of fixed (octameric) proportions. Instead, model archaeal histones oligomerize to form “hypernucleosomes”, flexible dimer stacks of variable size, wrapping an extra 30 bp of DNA with each dimer added to the stack (Maruyama et al. 2013; Mattiroli et al. 2017; Bowerman et al. 2021). Finally, model archaeal histones do not bear tails (Henneman et al. 2018) and evidence for PTMs, directed at the core fold, is—thus far—limited (Grau-Bové et al. 2022; Stevens and Warnecke 2022).

The evolution of eukaryotic nucleosomes, therefore, required the emergence of (a) distinct histone paralogs that carry (b) tails and exhibit (c) obligate and specific heterodimerization, and (d) assemble into a fixed octameric complex. Below, we will look for these features in archaea. Rather than conducting a broad survey of histone diversity across the entire domain Archaea, we will focus on the phylum thought to include the closest relatives of eukaryotes, the Asgardarchaeota (Box 1). The reason is simple: Finding some interesting feature, such as tails, in an archaeal lineage with only remote phylogenetic links to eukaryotes has no direct bearing on what happened during eukaryotic evolution. Looking at those archaea most likely to have contributed to the earliest eukaryotic lineages, on the other hand, can help us confirm, discard, or usefully constrain specific evolutionary scenarios. Importantly, however, looking at Asgardarchaeota by no means guarantees a direct line of descent between a feature found in these archaea and extant eukaryotes, as we will illustrate below.

Box 1. **Asgardarchaea—the closest archaeal relatives of eukaryotes.**

When it comes to reconstructing deep evolutionary relationships, one of the key discoveries of the last decade was that eukaryotes do not branch outside but inside archaeal diversity (Fig. A), challenging the three-domain model of evolution where all archaea share a common ancestor to the exclusion of eukaryotes (Williams et al. 2020; Eme et al. 2023, 2017).

**Fig. A.**
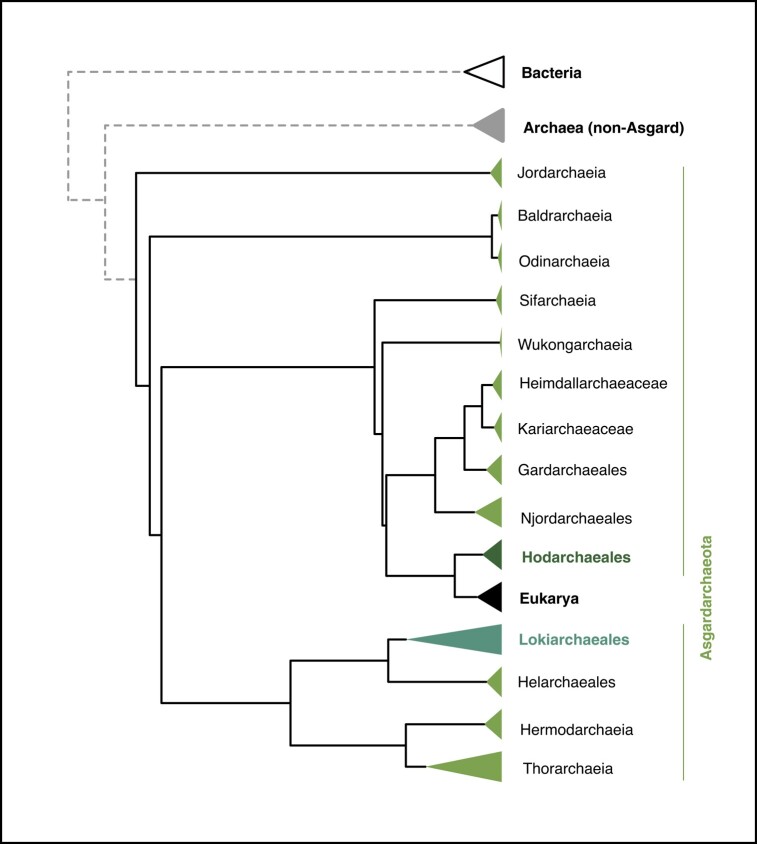
Phylogram highlighting the position of eukaryotes within the Asgardarchaeota. Adapted from Eme et al. (2023); based on amino acid tree NM-A175-nDK_sr4.CATGTR.c1.treelist.select.33.1000.tmp.contree available at https://doi.org/10.6084/m9.figshare.22678789. Dashed branches (leading to non-Asgard archaea and Bacteria) were added manually to indicate their relative phylogenetic position but are not to scale.

More sensitive and wider sampling of archaeal diversity using metagenomic approaches led to the discovery of the Asgardarchaeota, a cosmopolitan phylum of archaea found in a variety of marine and terrestrial environments (MacLeod et al. 2019). Reconstructing the phylogenetic relationships of genes found in both archaea and eukaryotes revealed that asgardarchaeal homologs—more often than not—branch with eukaryotes, rather than other archaea. Although it is worth remembering that genomes in general—and microbial genomes in particular—are mosaics where not all genes trace their ancestry to the same origin, repeated re-analyses and a stream of new data have lent support to the notion that the evolutionary proximity of Asgardarchaeota to eukaryotes is robust (Williams et al. 2020; Liu et al. 2021b; Eme et al. 2023). Recent and earlier work further highlighted the presence of “eukaryotic signature proteins”, genes commonly found in eukaryotes but not archaea—with the exception of Asgardarchaeota (Spang et al. 2015; Eme et al. 2017; Zaremba-Niedzwiedzka et al. 2017). At the same time, asgardarchaeal genomes very much follow a prokaryotic building plan in being organized into circular replicons that are considerably smaller than the genomes of most eukaryotes. What we know about the lifestyles and capabilities of these archaea is mostly derived from analyses of their genome content, which allows predictions to be made regarding, in particular, their metabolic capacities. Excitingly, two representatives of the Asgardarchaeota have recently been grown to high relative abundance in enrichment cultures (Imachi et al. 2020; Rodrigues-Oliveira et al. 2023). Functional data elucidating their chromatin structure and composition are not yet available, but appear within reach.

### Key Transitions I—Paralogs with Distinct Identities

An elemental step for the evolution of the nucleosome as we know it is the emergence of H3, H4, H2A, and H2B as distinct entities.

**Fig. 2.**
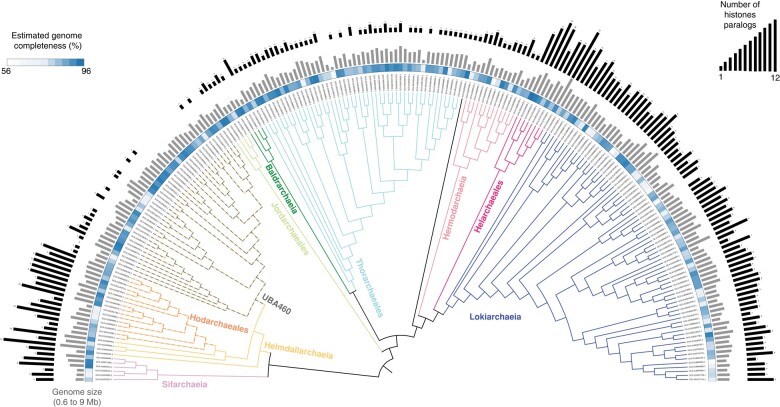
Variation in histone content across the Asgardarchaeota. Cladogram of the phylum Asgardarchaeota. Tree topology, genome sizes, and estimated genome completeness score (CheckM) are taken from GTDB (Parks et al. 2021) (https://gtdb.ecogenomic.org/). Orders within the class Heimdallarchaeia that are specifically named in the main text (UBA460, Hodarchaeales) are highlighted by dashed lines. Proteins with histone-fold domains were identified as detailed here: https://figshare.com/s/fbbba7dc93656d7cc495.

**Fig. 3.**
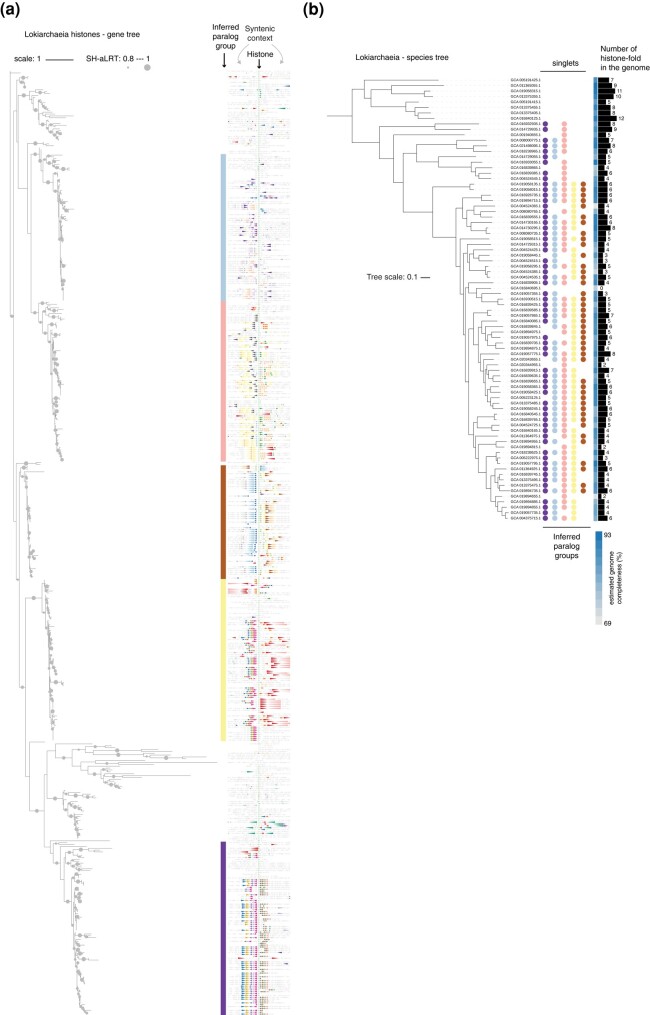
Conserved histone paralog in the Lokiarchaeia. a) Maximum likelihood protein tree of proteins with histone-fold domains in the Lokiarchaeia. Histone-fold proteins were identified and trees built using IQ-tree (Minh et al. 2020). Branch support values are derived from SH-like approximate likelihood ratio tests (SH-aLRT) as implemented in IQ-tree. Paralog groups were inferred from branching patterns in the tree and genomic context (synteny), which is displayed on the right, centered on the histone, with 10 genes upstream and downstream, represented as colored triangles. To highlight conserved neighboring genes, we pooled all proteins flanking the focal histones within a five gene range and clustered those proteins using mmseq2. Proteins belonging to the 30 largest clusters are colored. Methodological details, tree files, genome builds, etc. are available from FigShare (https://figshare.com/s/fbbba7dc93656d7cc495). b) Distribution of inferred histone paralog groups on the species tree of Lokiarchaeia. Tree topology and estimated genome completeness scores (CheckM) are taken from GTDB (Parks et al. 2021).

Archaea often encode two or three, and sometimes four or more, nonidentical histones (Stevens and Warnecke 2022). In some instances, paralogs with distinct sequences and properties have been maintained through hundreds of millions of years of evolution (Stevens et al. 2020, 2021; Stevens and Warnecke 2022). Although it remains unknown how these paralogs are deployed in genome regulation, long-term persistence of paralogs strongly suggests that adaptive functional diversification of histones occurred multiple times outside eukaryotes. Some members of the Asgardarchaeota are particularly rich in histone paralogs (Fig. 2); the cultured representatives, *Candidatus* Lokiarchaeum ossiferum and *Candidatus* Prometheoarchaeum syntrophicum (see Box 1), encode nine and seven histone paralogs, respectively. Both of these candidate species belong to the class Lokiarchaeia. Across most sequenced genomes in this clade, there are—based on phylogeny and synteny patterns—at least five recognizable paralogous groups, i.e. histone paralogs that were present in the ancestor of Lokiarchaeia and were inherited and maintained along different lineages within this group (Fig. 3).

It is worth noting, however, that histone paralogs are not universally numerous in all Asgardarchaeaota. Genomes in the order UBA460 (class Heimdallarchaea), for example, encode few, if any histones (Fig. 2). Asgardarchaeota, rather than signifying an inevitable march toward greater paralog complexity, are therefore perhaps better characterized as actively experimenting with their histone repertoires.

**Fig. 4.**
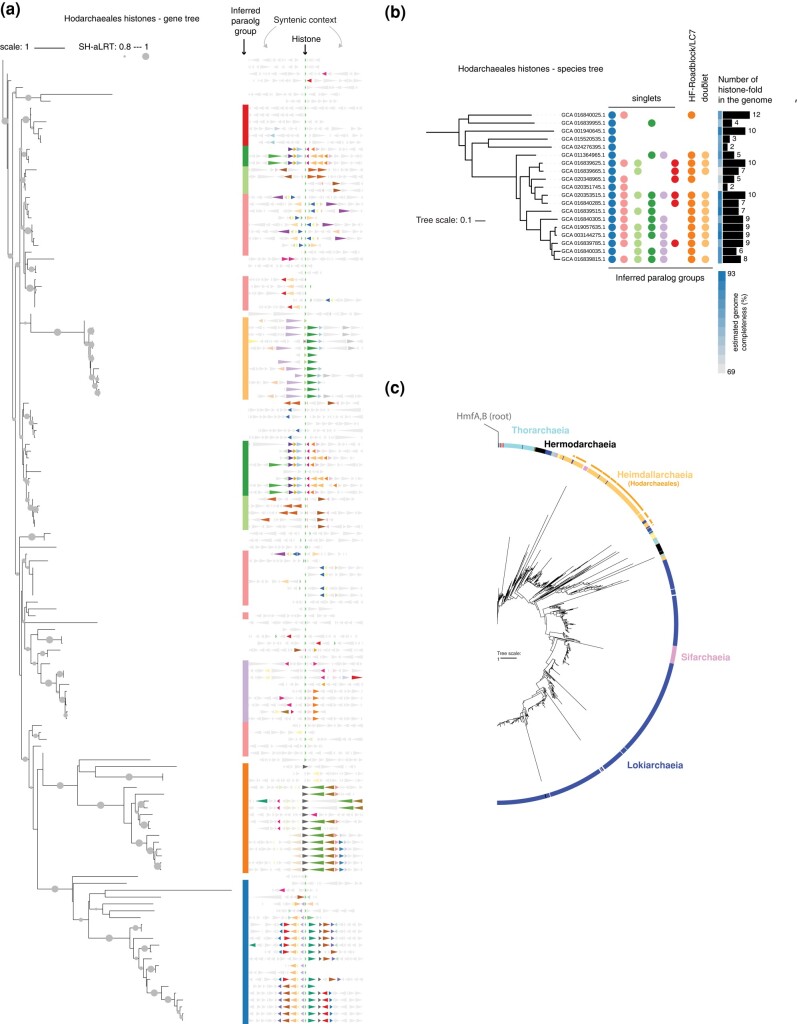
Conserved histone paralogs in the Hodarchaeales. a) Maximum likelihood tree of proteins with histone-fold domains in the Hodarchaeales. Histone-fold proteins were identified and the tree built using IQ-tree (Minh et al. 2020). Branch support values are derived from SH-like approximate likelihood ratio tests (SH-aLRT) as implemented in IQ-tree. Paralog groups were inferred from branching patterns in the tree and genomic context (synteny), which is displayed on the right, centered on the histone, with 10 genes upstream and downstream, represented as colored triangles. To highlight conserved neighboring genes, we pooled all proteins flanking the focal histones within a five gene range and clustered those proteins using mmseq2. Proteins belonging to the 30 largest clusters are colored. Methodological details, tree files, genome builds, etc. are available from FigShare (https://figshare.com/s/fbbba7dc93656d7cc495). b) Distribution of inferred histone paralog groups on the Hodarchaeales species tree. Tree topology and estimated genome completeness scores (CheckM) are taken from GTDB (Parks et al. 2021). c) Maximum likelihood tree of proteins with histone-fold domains in the Asgardarchaeota, built using IQ-tree and rooted using the euryarchaeal histones from *M. fervidus* (HMfA, HMfB) as an outgroup.

The Asgard clade with the closest phylogenetic affinity to eukaryotes, the Hodarchaeales (Box 1), encode seven histone-fold proteins on average (based on 20 sequenced genomes in GTDB, Parks et al. 2021). Based on their phylogenetic distribution and synteny, some paralogs are recent, lineage-specific additions, yet others date back to at least the root of the Hodarchaeales (Fig. 4a and b). Importantly, however, on a phylogenetic tree of all Asgardarchaeal histones, Hodarchaeales histones are monophyletic, as are histones from the Lokiarchaeia (Fig. 4c). This is consistent with independent histone diversification events in those two clades, rather than ancient paralogy. In other words, the set of histone paralogs in Lokiarchaeia and the set in Hodarchaeales are not derived from a shared ancestral set. This should be kept firmly in mind when using Lokiarchaeia in an attempt to shed light on the evolution of eukaryotic chromatin.

Most of the histone-fold proteins in the Hodarchaeales are similar to those found in model archaea, albeit slightly longer (hodarchaeal monomeric histones average 73 amino acids, while *T. kodakarensis* HTkA is 67 amino acids in length). But genomes in this clade also contain histones with longer tails, proteins where the histone fold is fused to a Roadblock/LC7 domain, thought to be involved in NTPase regulation in archaea (Koonin and Aravind 2000), and—as further discussed below—histone doublets (tandem fusion of histone folds), which are not seen in any Lokiarchaeia sequenced to date (Fig. 4b). We know nothing about any of these proteins in functional terms.

Is the histone diversity we see in the Hodarchaeales in any way related to the core histone classes present in LECA? In other words, did identifiable ancestors of H3, H4, H2B, and H2A (and H2A.Z, see Summary and Conclusions below) already exist in this clade? Or was ancient archaeal paralog diversity lost and eukaryotic paralogs diversified de novo at the time of (or at some point after) eukaryogenesis? A recent large-scale effort to reconstruct histone evolution found eukaryotic histones branching together outside the Asgardarchaeota, consistent with late diversification of eukaryotic histones (Irwin and Richards 2023). However, this analysis contained no histones from the Hodarchaeales. Consequently, how eukaryotic core histones relate to asgardarchaeal diversity remains an open question that should be regularly re-evaluated with the latest data.

### Key Transitions II—Obligate and Specific Heterodimerization

Eukaryotic histones have strict rules of association. In contrast, obligate and specific heterodimerization is not known for model archaeal histones, which readily homo- as well as heterodimerize (Sandman et al. 1994; Stevens and Warnecke 2022). To our knowledge, there is currently not a single known case of obligate and specific heterodimerization—inside or outside the Asgardarchaeota—although such cases might well be discovered in the future.

One potential pathway to obligate and specific heterodimerization is through physical shackling, where two histones are encoded as part of the same polypeptide chain. This shackling, usually through a short but flexible linker peptide, should strongly favor intramolecular dimerization. There are other benefits: rapid availability of a dimerization partner might facilitate solubility and guarantees stoichiometry of interaction partners without necessitating coordinated regulation. It might also explain the evolution of specificity in heterodimerization: when N- and C-terminal halves of these histone doublets diverged, they would have been under selection to maintain interactions with each other but not with other histones expressed in the same cell. Over time, perhaps simply through drift, those shackled histone folds might have lost the ability to dimerize with any other histone except their long-term forcible companions. The discovery of such histone doublets in archaea thus prompted the hypothesis that eukaryotic heterodimerization partners (H3–H4, H2A–H2B) might have evolved through a tethered intermediate whose shackles were ultimately broken (Malik and Henikoff 2003).

Histone doublets appear to have evolved multiple times independently in archaea as well as bacteria (Stevens et al. 2021; Hocher et al. 2023; Schwab et al. 2023). They are particularly common in Halobacteria but can also be found, for example, in the order Methanopyrales (Malik and Henikoff 2003; Stevens et al. 2021). Repeated de novo evolution of this domain architecture seems a reasonable proposition. As histones dimerize in a head-to-tail orientation, little more is required than a tandem duplication of a small protein (the histone) that generated a suitable linker. What argued against histone doublets as an intermediate on the path to eukaryotic heterodimer pairs was their absence in Lokiarchaeales—the first Asgard clade to be described and on which much subsequent analysis has focused. Intriguingly, as mentioned above, surveying the greater diversity of Asgardarchaeota genomes now available, one *can* find histone doublets—in the Hodarchaeales (Fig. 4b). Unlike some of the other histone doublets present in archaea (notably those annotated as having domain of unknown function DUF1931), the Hodarchaeales doublet has a high pI, suggesting that it may bind DNA. This finding offers no direct support for doublets as key intermediates on the way to the eukaryotic nucleosome, but it does illustrate that the Hodarchaeales were experimenting with histone fusions, making it more likely that such fusions were present in the earliest eukaryote.

More direct support for a fusion intermediate in the evolution of the eukaryotic nucleosome has come from a less obvious source: viruses. Specifically, viruses from the phylum Nucleocytoviricota (also known as nucleocytoplasmic large DNA viruses, NCLDVs). Some of these viruses encode free-standing histone proteins that were likely poached from their hosts in the recent past (Talbert et al. 2022; Irwin and Richards 2023). Others, however, encode a complete suit of core histone homologs (i.e. one H2A-like copy, one H2B-like copy, etc.), often fused into polypeptide chains that encode two, three or even all four core histone (Talbert et al. 2022; Irwin and Richards 2023). Recent structural work on viruses from the family Marseilleviridae, where the four histones are split across two doublets, has demonstrated that they form nucleosomal structures with striking structural homology to eukaryotic nucleosomes (Valencia-Sánchez et al. 2021; Liu et al. 2021a). More remarkably still, these viral histones are not just core histone look-alikes. As first noted by Erives (2017), when NCLDV histones are incorporated into phylogenetic trees alongside eukaryotic and archaeal histones, viral H4s branch with but outside of eukaryotic H4 diversity, viral H3s branch with but outside eukaryotic H3 diversity, and so on, rather than branching with the corresponding core histone of their known hosts (amoebae). This pattern led to the proposal that these viral histones are living relics that were picked up from an early (“proto”-) eukaryote, prior to LECA and the diversification of major eukaryotic lineages (Erives 2017). Recent comprehensive analyses involving many more viral genomes have reinforced this view (Irwin and Richards 2023; Karki and Aylward 2023) How long after the first eukaryotic common ancestor this histone acquisition process started and how long it continued before LECA remain open questions. What we know for sure is that, by the time of LECA, histones had split—irreversibly, it seems—into four monomeric units.

While viral histone sequence have provided an unexpected window into the deep eukaryotic past, the earliest events of nucleosome evolution remain mysterious. The Nucleocytoviricota encode full histone quartets, but how did we end up with four distinct core histones that assemble into an octameric complex? Did the earliest eukaryotic ancestor inherit the classic histone quartet (as fusions or monomers) from its archaeal predecessor? Or did this unique combination evolve during or soon after eukaryogenesis (but prior to LECA)? As we discussed in the subsection ‘Key Transitions I’, we cannot rule out either scenario at this point. Although fusions that involve more than two histones are not known from archaea, it would be premature—given the level of evolutionary experimentation evident in the Asgardarchaeota—to rule out that such constellations might yet be found. At the same time, it is perfectly possible that a single histone gene in the earliest eukaryote diversified, likely through fusion intermediates that facilitated complex assembly (Irwin and Richards 2023), into H2A, H2B, H3, and H4. This period of history, at present, remains beyond our empirical grasp.

### Key Transitions III—Tails: Easy Come, Easy Go?

Fusions are not the only mechanism to bring about obligate and specific heterodimerization. Another, perhaps less obvious way to enforce heterodimerization is growing a tail.

Tails are amongst the most talked-about features of eukaryotic histones, owing to their prominent role in signaling. While PTMs can be made to the histone fold itself (Tessarz and Kouzarides 2014), most signaling runs through residues in the tails, whose accessibility—jutting out from the tightly packed histone–DNA complex—enables histones to be modified without dismantling the nucleosome.

Recent work by Zhao and colleagues, using both structural modeling and molecular dynamics simulations, suggested that tails might also play a key role in biasing the choice of dimerization partners towards heterospecific histones (Zhao et al. 2023). Using human H2A as a case study, the authors found that the H2A core fold, stripped of its N- and C-terminal appendages, can form stable homodimers. Addition of tails interfered with H2A homodimerization but did not affect the formation of H2A–H2B heterodimers and appears instead to accelerate the folding transition to the dimer (Placek and Gloss 2005). Thus, the emergence of tails in one paralog might have been sufficient to trigger heterodimerization.

Though tails are overall rare in archaea, they are notably present in some Asgardarchaeota (Henneman et al. 2018; Eme et al. 2023), including some Hodarchaeales (see section ‘Key Transitions I’). Does this imply that the first eukaryotic common ancestor started its life with (some) tailed histones? Not necessarily.

**Fig. 5.**
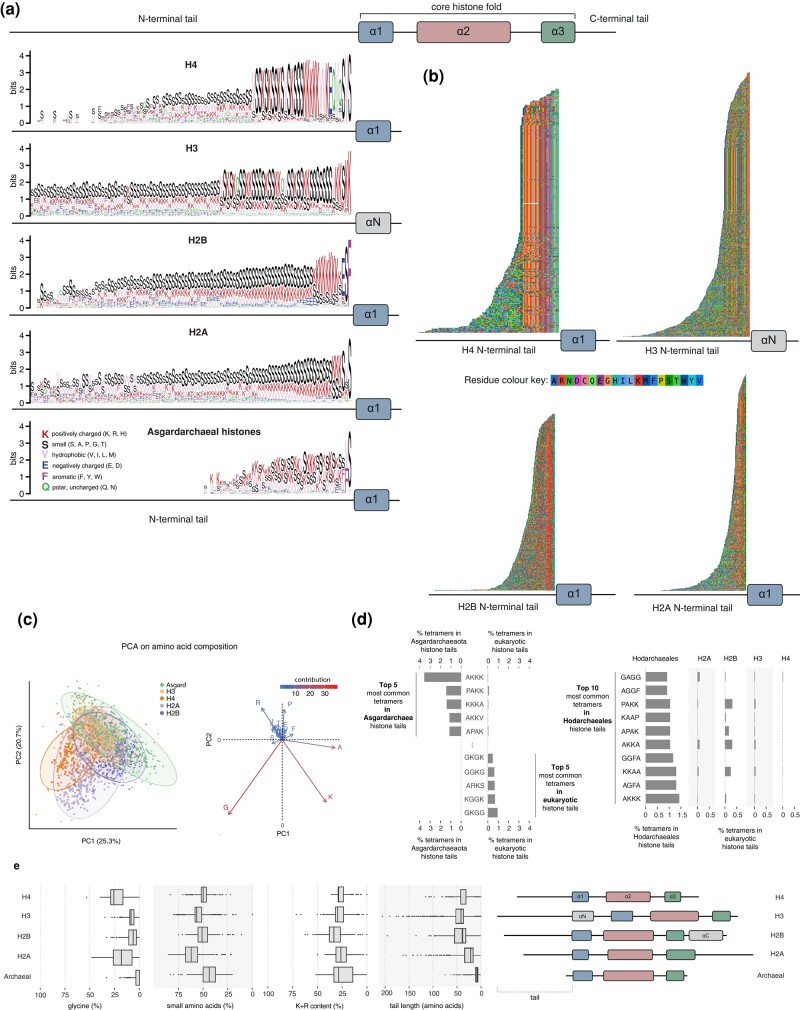
Comparison of eukaryotic and archaeal histones tails. a) Weblogos of histone tail sequences, recoded using the Dayhoff-6 scheme. Amino acid residues were recoded to highlight the relative abundance of biochemical properties rather than particular residues. Positions are defined based not on an alignment but on absolute distance from the first residue of the first alpha helix (α1 or in the case of H3, αN). Up to a maximum of 100 tail residues are shown. b) Tail sequences underlying the weblogos in panel (a) ordered according to their length, and starting from the first residue of the first alpha helix, but not aligned. Conservation of amino acids as a function of distance from the histone core fold is clearly evident. Note in particular the robust patterning of positively charged amino acids (K, R: red) and glycine (orange). c) Principal component analysis (PCA) on histone tails, as represented by relative amino acid frequencies. d) Amino acid tetramer signature across tails from different histone classes. K-mer frequencies were computed using a sliding window across all tails in a given class. e) Properties of asgardarchaeal and eukaryotic histone tails and the definition of tails in each class based on histone secondary structure. Note that no asgardarchaeal histone possesses a conserved N- or C-terminal helix as found in H3 and H2B, respectively. All sequences used in these analyses are available from FigShare (https://figshare.com/s/fbbba7dc93656d7cc495).

First, tails of each of the four core histone classes in eukaryotes are overall very well conserved, especially proximal to the histone-fold domain (Fig. 5a and b). As a consequence, it is easy to distinguish tails from different core histone classes from each other, as well as from tails in the Asgardarchaea. In fact, there is no discernible homology between eukaryotic and asgardarchaeal tails. The latter are also much shorter (Fig. 5e), with ramifications for the overall charge they can carry and thus their likely functional properties.

Second, disordered regions in proteins can lack obvious homology but exhibit strong conservation of short linear motifs (Sangster et al. 2022) or physicochemical properties (Zarin et al. 2019). Is this true here? Yes and no. At the most basic compositional level, there are some physicochemical similarities. Like their eukaryotic counterparts, asgardarchaeal histone tails are predicted to be disordered, carry an overall net positive charge, and alternate between small residues and positively charged residues, reminiscent of H3 tails (Fig. 5a to c). Further, in both Asgardarchaea and eukaryotes, the positive charges are primarily contributed by lysines whereas histidines are largely avoided, perhaps because the latter might negatively impact repair of abasic sites (Yang and Greenberg 2019). But look more closely and differences start to emerge: asgardarchaeal tails harbor, in proportional terms, notably fewer small amino acids as well as fewer glycines (Fig. 5e). At higher levels of complexity, the differences are undeniable. The most common amino acid tri- and tetramers in eukaryotic histone tails are rarely, if ever, found in Asgardarchaea, and vice versa (Fig. 5d).

The third reason why tails in Asgardarchaea need not be linked to eukaryotic histone tails is that evolving (and losing) a tail might be relatively straightforward. Histones in archaea (including in the Asgararchaeota) are usually encoded as single-gene operons (Hocher et al. 2022). All that is required, in principle, is a new upstream start codon (or downstream stop codon, for C-terminal tails) and a fortuitous lack of premature stops. As promoter/intergenic regions trend towards higher AT content in many microbial genomes, the result of extending the open reading frame might just be a tail rich in lysines (encoded by one of two codons: AAA or AAG). Several NAPs in prokaryotes, including bacterial histones in some Leptospira species (Hocher et al. 2023), and hupB in *Mycobacterium smegmatis* (Hołówka et al. 2017), probably gained tails in this manner.

Finally, it is worth noting that the fused Nucleocytoviricota histones discussed above largely lack tails (Irwin and Richards 2023), which might have been difficult to accommodate internal to fusion polypeptides. In so far as these viral histones provide an accurate impression of the past, that past would have been tailless. By implication, modern eukaryotic tails would have evolved comparatively late, only after pre-LECA histones had split into individual units, at which point the tails might have helped lock those units into a monomeric fate (Irwin and Richards 2023).

With no clear evidence of homology, a straightforward pathway for de novo evolution of tails, a lack of tails in NCLDVs, and physicochemical properties that are similar only at the lowest level of complexity, we think that, when it comes to understanding eukaryotic tails, asgardarchaeal tails might be somewhat of a red herring. At best, those small tails—if they existed in a direct archaeal ancestor of eukaryotes—could have provided an evolutionary foothold that allowed further elaboration into longer tails, now unrecognizable from their ancestral starting point. Alternatively, the tails we see in Asgardarchaeota today represent instances of parallel evolution entirely unrelated to eukaryotic tails.

### Properties of the Ancestral Nucleosome

Thus far, we have been concerned with the evolutionary history of the nucleosome as a complex. To gain a full understanding of its evolution, we also need to consider the evolution of its individual components, the four core histones, at the peptide level. This will be critical to address questions that go beyond whether the nucleosome existed toward what properties nucleosomes had at a given point in the past. As we saw above, considerable uncertainty surrounds the initial formation of the nucleosome in the earliest (pre-LECA) eukaryote so an evaluation of the sequence features of its individual components is currently not possible. The same, however, might not be true for nucleosomes at the time of LECA. At that point, the nucleosome existed in more or less its current form. How do the properties of the LECA nucleosome compare to that of extant nucleosomes? Did the octameric histone complex bind DNA as strongly as in, say, a human nucleosome? Was the complex more or less stable than what we find in the present? Was the acidic patch, which plays a key role in mediating interactions between neighboring nucleosomes, established in its current form? Answers to these questions are important because they might tell us something about genome accessibility in LECA, with implications for understanding the evolution of transcriptional ground states (Struhl 1999). Viral histone sequences, as useful as they have proven for understanding the evolution of the nucleosome as a complex, are of limited value here. Viral histones might be relics but they are not frozen in time. They have continued to evolve from the time when they were picked up from an early, proto-eukaryotic lineage, and have likely done so under considerably different selection pressures from those that have affected histones that package eukaryotic genomes. They are no closer to LECA than the average eukaryotic histone in phylogenetic terms (and often considerably more diverged at the amino acid level), and thus provide no direct window into the properties of the ancestral nucleosome.

Instead, given the exceptional levels of conservation of histones compared to other proteins, we might gain valuable insights into nucleosomes past via ancestral sequence reconstruction. Attempts in that direction have been surprisingly limited, with the exception of two decade-old independent efforts to reconstruct LECA H3 (Postberg et al. 2010; Waterborg 2012). Both yielded a mostly well-defined ancestral state, with >85% identity to human H3.1. More suitable models of sequence evolution and improved coverage of extant eukaryotic diversity might help to fully define not only LECA H3, but also H4 (the most highly conserved histone), H2A, and H2B (the least conserved). Even if divergence in parts of the histones, notably the tails, proves sufficient to thwart alignment-based ancestral reconstruction, it might nonetheless be possible to capture relevant ancestral states, by focusing exploration on conserved short linear motifs and/or broader molecular features (e.g. hydrophobicity), which often remain conserved even where primary sequences do not (Zarin et al. 2019; Sangster et al. 2022). Reconstructed ancestral sequences could then be characterized with the diverse toolkit available to chromatin afficionados, including in vitro, in vivo, and in silico approaches.

## Summary and Conclusions

We have examined a number of key transitions required for the emergence of the nucleosome: the evolution of distinct paralogs, obligate and specific heterodimerization, and the acquisition of tails. For each of these features there is precedent in the evolutionary history of archaeal and/or viral histones: Several archaeal lineages evolved paralogs with distinct properties that have been adaptively retained over time. Some archaeal histones grew tails including, intriguingly, some histones in the Hodarchaeales. And while we lack evidence for obligate heterodimerization of monomeric archaeal histones, the existence of doublets (in archaea), triplets, or even quadruplets (in Nucleocytoviricota) provides a credible evolutionary path towards such a fate. All these steps, therefore, are plausible. But while we can see that the closest living archaeal relatives of eukaryotes have been experimenting with histones in apparently pertinent ways, this has not (yet?) led us to discover direct evolutionary intermediates. There are tails, but while biochemically somewhat reminiscent of histone tails in eukaryotes, they are not obviously homologous. There are paralogs and histone fusions in archaea but there is no evidence to date that these trace forward through evolution to the four eukaryotic core histones. This critical period, somewhere around the point of eukaryogenesis but with a wide margin of temporal error, when ancient cells settled on the quaternity of core histones and a fixed octameric architecture, is poorly resolved in terms of molecular evolution. We emerge from the darkness thanks to the living fossil record of viral genomes, which suggests a period of histone fission and fusion in early eukaryotic lineage(s), before histone concatenates eventually split into four monomeric units. This split was well established by the time of LECA; an inference that does not solely rest on the observation that—as a rule—current eukaryotes do not have fused histones. It is also consistent with the observation that at least one histone variant, H2A.Z, appears to trace its roots all the way back to LECA (Malik and Henikoff 2003). For histone variants to exist as part of the nucleosome, monomeric interaction partners need also exist. How else would variants be swapped in and out?

Tails too were firmly established at that point as key features of histone biology. Again, this is not only based on homology and the unusual conservation of tails across eukaryotes (Fig. 5b). It is also evident from reconstructing the wider repertoire of LECA proteins, which includes a large number of histone PTM readers, writers, and erasers as well as proteins involved in nucleosome remodeling (Grau-Bové et al. 2022).

What discoveries might advance our understanding of nucleosome evolution in the future? A thorough investigation of the most recent asgardarchaeal histone sequences and their relationship to the four eukaryotic histones is overdue and might reveal ancestral paralogy that spans the two domains, uncovering the molecular Archaeopteryx that provides the elusive link between archaeal and eukaryotic histones. One ought to brace, however, for the possibility that such evidence has been lost to history. Finally, functional dissection of lokiarchaeal histones in their native context is inching ever closer. If not directly informative of the formative years of the nucleosome (see section ‘Key Transitions I’), functional studies have the capacity to reveal what roles histones played prior to eukaryogenesis, what selective forces were acting on histones before the nucleosome arose, and what might have driven the emergence of salient features such as histone tails.

## Data Availability

There are no new data associated with this article.
